# 354. A Comparison of Different Strategies for Optimizing the Selection of Empiric Antibiotic Therapy for Hospital-Acquired Pneumonia (HAP) and Ventilator-Associated Pneumonia (VAP) Caused by Gram-Negative Bacteria in Intensive Care Units (ICU)

**DOI:** 10.1093/ofid/ofad500.425

**Published:** 2023-11-27

**Authors:** Walaiporn Wangchinda, Samuel L Aitken, Paul Lephart, jason M Pogue

**Affiliations:** University of Michigan College of Pharmacy, Ann Arbor, MI; Michigan Medicine, Ann Arbor, Michigan; University of Michigan, Ann Arbor, Michigan; University of Michigan, College of Pharmacy, Ann Arbor, Michigan

## Abstract

**Background:**

Guidelines for HAP/VAP recommend considering local susceptibility data and patient specific risk factors for empiric therapy. However, data are lacking assessing whether a unit-specific combination antibiogram or a patient-specific factor-based approach will better drive appropriate empiric treatment. This study aims to hypothetically compare the two methods.

**Methods:**

This retrospective study was conducted at Michigan Medicine between 2021-2022. Microbiological data of 221 non-duplicated, first Gram-negative respiratory isolates obtained from 190 patients in the MICU and SICU in 2021 were used to develop unit-specific combination antibiograms (Table 1) and individual patient charts were accessed to identify the impact of the presence/absence of various risk factors on antimicrobial susceptibility to define optimal regimens. Selected empiric regimens based on each approach are shown in Table 2. These two approaches were then hypothetically applied to 129 patients with positive respiratory cultures in 2022. The appropriateness, defined by having at least one *in vitro* active agent, and the overuse of antibiotics in empiric regimens by the two approaches were then assessed and compared.

**Figure:**
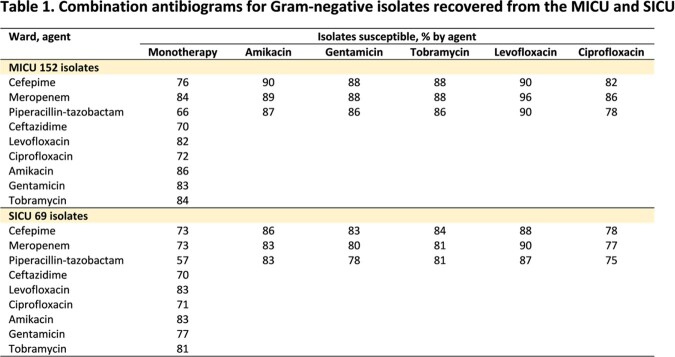

**Figure:**
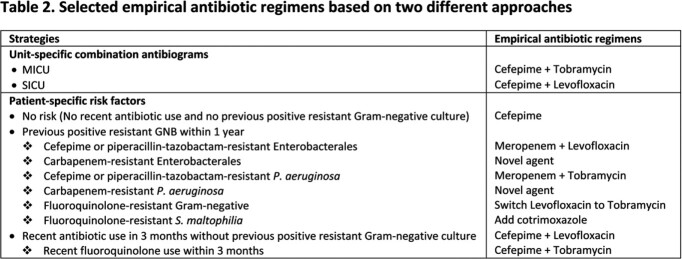

**Results:**

The breakdown of different empiric regimens selected based on the two approaches are shown in Table 3. Cefepime was the backbone β-lactam in regimens for both MICU and SICU by unit-specific combination antibiograms and in 83.7% of regimens based on patient-specific risk factors. Empiric regimens based on patient-specific risk factor had a higher rate of appropriateness than those based on antibiograms (89.1% vs 83.7%), with a 33.1% reduction in the rate of inappropriate empiric therapy. Additionally, using combination antibiograms resulted in a higher prevalence of antibiotic overuse than using patient-specific risk factors (72.9% vs. 40.3%), with most inappropriate use due to unnecessary aminoglycoside use. The overuse of other agents was comparable between approaches.

**Figure:**
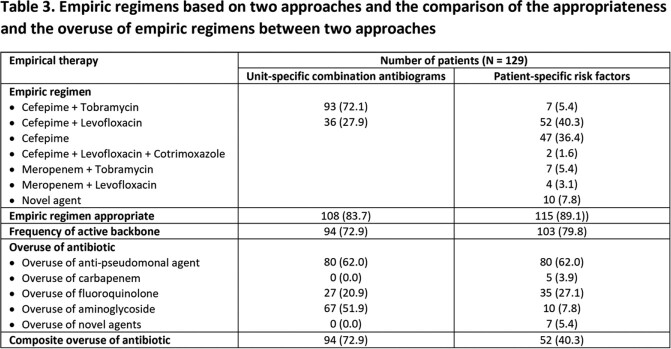

**Conclusion:**

Both approaches were useful and can be applied in empiric antibiotic selection. However, the patient-specific risk factor-based approach offers advantages in reducing rates of inappropriate therapy and decreasing the overuse of combination agents.

**Disclosures:**

**jason M. Pogue, PharmD**, AbbVie: Advisor/Consultant|Entasis: Advisor/Consultant|Ferring: Advisor/Consultant|GSK: Advisor/Consultant|Merck: Advisor/Consultant|Merck: Grant/Research Support|Qpex: Advisor/Consultant|Shionogi: Advisor/Consultant

